# Health Policies in Romania to Reduce the Mortality Caused by Cardiovascular Diseases

**DOI:** 10.3390/ijerph16173080

**Published:** 2019-08-24

**Authors:** Mihaela Simionescu, Svitlana Bilan, Beata Gavurova, Elena-Nicoleta Bordea

**Affiliations:** 1 Institute for Economic Forecasting of the Romanian Academy, 050711 Bucharest, Romania; 2 Faculty of Management, Rzeszow University of Technology, 35-959 Rzeszów, Poland; 3 Research and Innovation Centre Bioinformatics, Technical University of Košice, USP TECHNICOM, 040 01 Košice, Slovakia; 4 Technical University of Košice, 040 01 Košice, Slovakia; 5 Faculty of Midwifery and Nursing, “Carol Davila” University of Medicine and Pharmacy, 020021 Bucharest, Romania

**Keywords:** heart diseases, healthcare policy, healthcare expenditures, cardiologist, hospital

## Abstract

Cardiovascular diseases (CVDs) are the main cause of death in Romania. The objective of the paper is to explain the number of deaths caused by the diseases of circulatory system in relation to health expenditures per capita, the number of public hospitals with cardiology sections and the number of cardiologists. The analysis in the paper is based on panel data models and Bayesian linear models. A panel data approach for Romanian macro-regions in the period from 1995–2016 was used to show that an increase in the number of cardiologists would reduce the number of deaths caused by the diseases of circulatory system. The Bayesian approach to national data indicated that the increase in expenditures per capita would alleviate the incidence of deaths caused by CVD. The public health policies in relation to patients with CVD should focus on the future on higher expenditures per person, but the number of public hospitals and doctors treating these diseases should also continue to grow. Future healthcare policies should be also focused on reducing the number of specialists migrating to wealthier countries.

## 1. Introduction

The European cardiovascular disease report 2017 shows that diseases of cardiovascular system are responsible for 1.8 million deaths in the European Union (EU) and cover the 37% share on total deaths in the EU countries [1]. According to the available evidence, death rates from both ischaemic heart disease (IHD) and strokes are generally higher in Central and Eastern Europe than in Northern, Southern and Western Europe. The rates of lives lost because of CVDs are also generally higher in Central and Eastern Europe than in Northern, Southern and Western Europe [1,2,3,4,5,6,7,8,9,10,11,12,13,14,15,16,17,18,19,20,21]. Overall, CVD is estimated to cost the EU economy €210 billion a year and of the total cost of CVD in the EU, around 53% (or €111 billion) are healthcare costs, 26% (€54 billion) are productivity losses and 21% (€45 billion) fall on the informal care of the people with CVD [20].

The European health statistics show [22,23,24] that 153,953 deaths can be accounted to CVD in Romania and these are 59.3% of all deaths in the country (2015). Among all deaths related to CVD, 52.8% are in the male population and 66.4% in the female. Standardized death rates from CVD in Romania [22] are 954.8 for both sexes, 1103.0 for male and 841.3 for female respectively (per 100,000 inhabitants). On the one hand, for persons aged less than 65 years, the standardized death rate was 114.6 and 140.423 for the people at the age of 65 and over (per 100,000 inhabitants). Generally, Romania has the worst indicators in this regard among the EU28 [22].

National Health Insurance House in Romania is the public institution that ensures unitary and coordinated functioning of the system of health social insurances. Its mission is to implement an efficient system of health insurance as to improve the health status of the population. It has to cover the needs for healthcare services within the limits of its budget (€0.2 million in 2019). The budget of this public institution represents around 4.8% of GDP. Even if cardiovascular disorders are the main cause of deaths in Romania, the government allocates them a sum which is 14 times less than the allocated sum for oncological diseases and 8 times less than the amount for dialysis. Treatment of the main mortality source in our country receives only 4.5% from the budget of the National Health Insurance House. This is only 0.002% of GDP. Moreover, the entire healthcare system suffers from underfinancing, because Romania is an economically weak performing country in comparison with some other EU countries. The reform in healthcare domain went in hand with political transformations after the communism fall back in 1989 [3,4,23]. Overall low level of GDP explains low budget of the public healthcare sector. Despite budget issues, public hospitals still have to be competitive and provide a quality of healthcare services similar to private hospitals [5]. Hospitals’ efficiency should be stimulated [6,7,8,9,10] through increases in budget allocations. Different strategies should be applied as to investing more in cardiology departments of public hospitals.

There are only four intervention centres in the country involved in the Acute Myocardial Infarction program for the settlements with a population of 2,500,000 people. Only one of them, the Institute of Cardiology, is public, the other three are private centres. New public centres should be built involving prestigious specialists in the field.

Romania is the second country in the European Union, after Bulgaria, according to the mortality rate. Life expectancy at birth is 73.8 years in Romania, with a higher value for females (77.5 years) as compared to males (70.1 years). According to the latest data, CVDs are the main cause of deaths in Romania (35% of the total deaths), being followed by tumors (12% of deaths), diseases of respiratory system, digestive system, infectious diseases, accidents, poisoning. Females’ mortality from CVDs is higher than that of males (53%–56% of the total deaths from CVDs fall on females), the same can be also said about endocrine diseases, nutrition and metabolism dysfunctions. Mortality rates among males are higher as compared to females in cases of tumors, poisoning, digestive and respiratory diseases [4].

CVDs are associated with such issues as smoking, hypertension, elevated cholesterol, obesity, diabetes, physical inactivity [2]. According to the data by the National Institute for Public Health in Romania, the most important risk factors in relation to CVDs are: hypertension (31.8% of the CVD-related deaths), tobacco (16.3% of CVD-related deaths), elevated cholesterol (14.4%), obesity (13.9%), alcohol (12.4%), low fruit and vegetable intake (7.1%) and physical inactivity (6.6%).

Hypertension is the most important factor in terms of risk of CVDs in Romania. High blood pressure is observed among 45.1% of all adult population in the country. Or in other words, 7.4 million people in Romania are affected by hypertension. Almost 30% of the Romanian population are smokers. The largest shares of smokers are observed in the following age groups: 15–24 years (34.8% of population) and 25–34 years (32.9% of population). Most of Romanian smokers (61.6%) consume between 10 and 20 cigarettes per day. The prevalence of diseases caused by alcohol is 2.4% in Romania. A total of 17,000 of Romanians die annually because of alcohol. Romania has the highest standardized rate of mortality in the European Union for females and is ranked as fourth in case of males [10].

The prevalence of obesity in Romania is around 20%, with higher incidence among males as compared to females. The rate of elevated cholesterol level is high in Romania, same applies to some other Eastern European countries like Ukraine, Bulgaria, Hungary, Lithuania, and Russia [2]. However, the rates of these countries are considered low if we take into account their healthcare systems and low development of their economies.

Forty-one percent of the Romanian population consume fruit and vegetables on a daily basis. The prevalence of physical inactivity is higher among women (45%) as compared to men (40%).

Considering CVDs as a priority for Romanian healthcare system because they are the most frequent causes of deaths, the aim of our research is to assess the impact of different factors on the occurrence of deaths caused by CVDs. This aim is achieved via two objectives: assessment of the impact of different factors on deaths caused by CVDs at the national level and assessment of the impact of different factors on deaths caused by CVDs at the regional level. The panel data approach applied in this study suggested there is an urgent need for cardiologists more than the necessity to build hospitals caring of the patients with heart diseases. The situation in Romania is similar to that in Sub-Saharan Africa when it comes to the number of doctors. Thus, in Romania, there are about 700,000 patients, which means 9000 patients per doctor, or 36 patients per day for a specialist in vascular surgery. In addition, less than 40% of children with cardiovascular diseases in Romania are operated on in the country because of the current situation with financing of healthcare programs.

## 2. Materials and Methods

The methods used in the empirical research are conditioned by the type of data. In this case, we used Bayesian linear regression models for time series at national level and panel data models for panel data at regional level.

The variables are represented by the number of deaths caused by CVDs, health expenditure per capita (in PPP constant 2011 international $), number of public hospitals where CVDs are treated, and number of cardiologists in these hospitals divided by the number of population in Romania. All the data are registered for Romania in the period from 1995–2016. The source of data for health expenditure per capita is Eurostat, while the data for the rest of the variables are provided by National Institute for Statistics through its online database, called Tempo online.

Bayesian linear regression models are used because of the short data set (time series for the period from 1995–2016). The panel data models are also used to eliminate the disadvantage of the short available data sets.

The Bayesian linear regression model is expressed as follows:

(1) $$Y_{t}=\beta \cdot X_{t}+e_{t}$$

$$\beta \sim N\left(m_{u},V\right)$$

$$s_{e}^{2}\sim IG\left(a,b\right)$$

*Y*—dependent variable (number of deaths caused by CVDs)
*X*—explanatory variables (regressors) (the rest of the variables)
β—vector of coefficients
$e_{t}$—error
$s_{e}^{2}$—variance of error
*n*—time series length
$m_{u},V$—parameters in the normal distribution ($m_{u}\;is\;fixed\;to\;1,V\;is\;the\;unit\;matrix)$
*a, b*—parameters in the inverse-gamma distribution

Conditional posterior of β has a normal distribution [10]. Conditional posterior of $s_{e}^{2}$ has an inverse-gamma distribution: $IG\sim \left(\frac{n}{2}+a,\frac{2b}{b\cdot RSS+2}\right).$ a and b are set up to value 1 by researchers based on own experience. *n* represents the number of observations (*n* = 12, as there are 12 years).

RSS—sum of square residuals.

The estimation method is represented by the Metropolis Hastings algorithm that is automatically implemented by the software (the calculations are made in the software STATA 15).

The methodology for panel data starts from a regression model based on cross-section data (4 cross-sections) and time series (period from 1995–2016). The number of observations in this case is calculated as follows: number of years x, number of cross-sections (22 × 4 = 88 values). The method called pooled ordinary least squares was used as the first one (11). The fixed-effect or random-effect panel data model may be represented as follows:

(2) $$y_{it}=\beta_{0}+\underset{j}{\sum }\beta_{j}X_{jit}+e_{it}$$

*i* = 1, 2, ..., N
*t* = 1, 2, ..., T
$y_{it}$—dependent variable on time *t* for cross-section *i* (number of deaths caused by CVDs)
$X_{jit}$—the *j* independent variable on time *t* for *i* unit (the rest of the variables)
$e_{it}$—error
$\beta_{j}$—coefficient or parameter
$\beta_{0}$—intercept

This basic model is transformed to use estimation methods, which are specific to panel data models. The fixed-effect model allows the individual effects. If the specific cross-section effect is constant in time, the unobserved characteristics are seen as fixed effects that have different values for each cross-section ($\beta_{0i}$) in the constant term of the model. The unobserved heterogeneity is controlled since it is constant in time and it may be eventually correlated with the regressors of the model. The one-way fixed-effect model has the following representation:

(3) $$y_{it}=\beta_{0i}+\underset{j}{\sum }\beta_{j}X_{jit}+e_{it}$$

*i* = 1, 2, ..., N
*t* = 1, 2, ..., T
$y_{it}$—dependent variable on time *t* for cross-section *i* (number of deaths caused by CVDs)
$X_{jit}$—the j-it independent variable on time *t* for cross-section *i* (the rest of the variables)
$e_{it}$—error
$\beta_{j}$—coefficient
$\beta_{0i}$—unobserved individual effect corresponding to cross-section *i*, which does not change in time (it includes cross-section fixed effects)

The two-way fixed-effect model is presented by considering the fixed effects to time dimension:

(4) $$y_{it}=\beta_{0i}+\gamma_{t}+\underset{j}{\sum }\beta_{j}X_{jit}+e_{it}$$

$\gamma_{t}$—fixed effects in time.

Firstly, it is possible to choose between the fixed-effect model and the pooled ordinary least squares model. F-test is applied to verify the presence or the absence of individual effects (12). The assumptions of the F-test are written as:

H0: $\beta_{0i}=0$ (absence of fixed effects)

H1: $\beta_{0i}\neq 0$ (presence of fixed effects)

The F-test uses this statistics:

(5) $$F=\frac{\frac{ESS_{POLS}-ESS_{FE}}{N-1}}{\frac{ESS_{FE}}{\left(T-1\right)N-K}}$$

*K*—number of regressors in the fixed-effect model
*N*—number of cross-sections
*T*—number of time periods
$ESS_{POLS}$—sum of square errors in case of pooled ordinary least squares model
$ESS_{FE}$—sum of square errors in case of fixed-effect model

Fixed-effect model has only individual constants, while random-effect model treats constant one as a random variable of average $\beta_{0}.$ Differences between cross-sections are considered to be random deviations from the average.

(6) $$\beta_{0i}=\beta_{0}+\varepsilon_{i}$$

$\varepsilon_{i}$ is the error with null average and constant variance ($\sigma_{\varepsilon }^{2}$).

The composite error is defined as:

(7) $$u_{it}=\varepsilon_{i}+e_{it}$$

$\varepsilon_{i}$—error for cross-sections
$e_{it}$—random error

The random-effect model is written as:

(8) $$y_{it}=\beta_{0}+\underset{j}{\sum }\beta_{j}X_{jit}+u_{it}$$

*i* = 1, 2, ..., N
*t* = 1, 2, ..., T

The Hausman test is used to decide between fixed-effect model and random-effect model.

## 3. Results

A Bayesian linear regression model was built to explain the number of deaths caused by CVDs in Romania. The explanatory variables are represented by health expenditure per capita (in PPP constant 2011 international $), number of public hospitals where CVD are treated, and number of cardiologists in these hospitals. All the data are registered for Romania in the period from 1995–2016. The Romanian population decreased by 15% in the period from 1995–2016, but the number of deaths caused by CVDs decreased in the same period only by 3% in case of females and by 8% in case of males. If we correlated the number of deaths caused by CVDs with the reduction of population, we may conclude that actually, the number of deaths caused by CVDs increased.

The short data set imposes the use of Bayesian techniques. The results of the Bayesian estimation using the Metropolis–Hastings algorithm are presented in Table 1.

According to the estimation results, the health expenditure per capita had a negative impact on the number of deaths. The increase in the health expenditure per person reduced the number of deaths causes by diseases of circulatory system in Romania. However, contrary to expectations, even if the number of doctors and hospitals is greater, the deaths continued to increase. This confirms our hypothesis that the number of hospitals and doctors treating heart diseases is still very low in Romania.

A panel data model was built to identify the determinants of deaths caused by heart diseases at regional level in Romania. The variables are represented by the number of deaths caused by diseases of circulatory system, number of cardiologists and number of public hospitals treating among other diseases the heart diseases. The data are collected for the period from 1995–2016 for the four macro-regions of Romania known as macro-region 1, macro-region 2, macro-region 3 and macro-region 4. A fixed-effect model in Table 2 described the relationship between cases of death caused by cardiovascular diseases and number of public hospital treating these affections and number of cardiologists.

An increase in the number of cardiologists by one doctor reduced, on average, the cases of death by almost one cases. The number of public hospitals treating cardiovascular diseases increased, but the cases of death caused by this health problem continued to increase. As we may observe, the results at regional level are different from those at national level for the influence of the number of cardiologists.

## 4. Discussion

The differences between the results at national level and those of regional level might be explained by the fact that there are high discrepancies between macro-regions regarding the number of doctors. Most of the cardiologists are concentrated in the public hospitals in the capital, while in poor and small cities fewer cardiologists might be found.

The public health policies in the field of patients with CVD problems should focus on the future on more expenditure per person, but the number of public hospitals and doctors treating these diseases should continue to increase.

Romania has experienced an intensive process of emigration among the young doctors because of the low salaries and high level of corruption in the system. The money used in the public health system for the people with heart diseases should be seen as a long-run investment and not a simple expenditure. A greater transparency is required in spending public money in the health sector, but also in the entire public sector. A priority for the health system should be the increase in the financing of the public health system, because the low level of the resources for health directly affects the quality of medical acts and it is an important cause of medical migration. The data provided by the College of Physicians indicated that the most departures of Romanian physicians are from main cities in the country, including the capital Bucharest. The most frequent destination countries are the United Kingdom, France, Germany, Spain, Italy, Austria, Belgium, Portugal, Cyprus, Netherlands and Canada. The most required specializations in these countries are family medicine, general medicine, intensive care, and anesthesia [13].

To date, 17,000 cardiovascular surgical procedures that should be performed, but the National Health Insurance House only finances 5600 of them. In West European countries, the average amount allocated to a surgical case is about € 18,000 while in Romania, the state allocates 10 times less (between € 1200–1800). Even Bulgaria and the Republic of Moldova provide the funding € 7000 per case.

The network of pediatric cardiologists is deficient in Romania and the specialty is completely ignored in this country. Many children die in the absence of intervention centres and many families have no money to finance the operation although they benefit from the provisions of Form E 112, through which the state can make a substantial contribution to cover the hospitalization costs outside the country.

We can conclude that more money should be allocated for the treatment of patients with cardiovascular diseases, money that include better salaries for cardiologists. A long-term strategy should be implemented to transform the cardiovascular disorders as a national priority in health domain. Practical solutions should be considered to limit the cases of death caused by these diseases: an equitable reallocation of funds in the curative programs for a non-discriminatory treatment of patients with cardiovascular disorders (poorer Romanians should not be discriminated against compared to richer ones), less discrimination for new medical centres by assurance system, the application of the principle of the Health Law, according to which the money follows the patient, thus offering the equitable access of the Romanian patients to all the medical structures in the field, without the Romanian patients insured to pay very large amounts for the right to life.

The public-private partnerships should intensify as these bring benefits first and foremost to patients, but also to the community and academia [14]. The role of vascular surgery increased in time since the number of procedures in this domain grew by four times in the last 10 years due to capacity building. Therefore, the attention assigned to these procedures should continue. Cardiovascular care has intensified, but efforts are still necessary to prevent cases of death. The public policies in Romania for preventing CVDs are related to promotion of information regarding healthy eating, physical activity, reduction of consumption of alcohol, sugar, salt and cigarettes. However, the partnership between life science sectors and health should be extended. Private and governmental agencies should cooperate in fields like education, culture, recreation, commerce, and agriculture. It is not enough to treat the diseases. Various measures should be considered to treat the causes and risk factors of CVD (behavioral determents of health). Notably, Romania ranks among the countries with the highest prevalence of smoking in the world. The country is located in the red zone of hypertension (HTA), in a very advanced area of diabetes prevalence and high cholesterol in the blood [15,16,17]. All of these conditions need to be address by specific Romanian goals in the framework of European Health 2020 strategy [25]. The lack of cardiologists in smaller centres could be overcome by improving the access to care in major cities: better support for families of patients receiving treatment outside of their home towns, patient transfers via helicopter, etc. Enhancing the coordination of patient care seems necessary as well.

## 5. Conclusions

Romania is the only country in Europe where life expectancy is continuously deteriorating. Almost 60% of the Romanians’ deaths are caused by cardiovascular diseases, the main reason being the unfair access of population to healthcare infrastructure and to advanced technologies in medicine [18]. Considering CVDs as a priority in future healthcare strategies in Romania, we assessed the impact of various factors on the cases of death caused by heart diseases. The Bayesian model based on empirical data of the national level suggests that the number of public hospitals and cardiologists are positively correlated with the cases of death because of cardiovascular diseases, unlike healthcare expenditure per capita. At the regional level, the panel data approach indicates that there are consistent discrepancies and the necessity for more cardiologists in small cities is more acute as compared to the capital of Romania. Overall, the empirical results indicate that an increase in the number of cardiologists in all Romanian macro-regions and higher healthcare expenditure per person at the national level has the potential to reduce the cases of death from cardiovascular disorders. Improvements in financing and quality of medical services, a non-discriminatory access of Romanian patients to the best methods and stopping the emigration of young doctors should be considered among the solutions for reducing the cases of death from cardiovascular diseases [19,20].

## Figures and Tables

**Table 1:** The impact of different factors on the deaths caused by CVDs in Romania (1995–2016).

Variable	Posterior Mean	Posterior Standard Deviation
**Constant**	0.1	10.0
**Health expenditure per capita**	−14.6	3.6
**Number of public hospitals**	0.3	9.1
**Number of cardiologists/number of population**	0.3	0.2
**Variance**	3.7	1.1

Source: own calculations.

**Table 2:** The impact of different factors on the deaths caused by CVDs in Romanian macro-regions (1995–2016).

Variable	Coefficient	t	P > |t|
**Constant**	317.8	7.8	0.0
**Number of public hospitals**	106.7	−0.8	0.0
**Number of cardiologists**	−0.3	1.8	0.0

Source: own calculations.
